# Unique Epigenetic Features of Ribosomal RNA Genes (rDNA) in Early Diverging Plants (Bryophytes)

**DOI:** 10.3389/fpls.2019.01066

**Published:** 2019-09-05

**Authors:** Roman Matyášek, Alice Krumpolcová, Jana Lunerová, Eva Mikulášková, Josep A. Rosselló, Aleš Kovařík

**Affiliations:** ^1^Department of Molecular Epigenetics, Institute of Biophysics of the Czech Academy of Sciences, Institute of Biophysics of the Czech Academy of Sciences, Brno, Czechia; ^2^Department of Botany and Zoology, Masaryk University, Brno, Czechia; ^3^Jardín Botánico, ICBiBE-Unidad Asociada CSIC, Universidad de Valencia, Valencia, Spain

**Keywords:** rDNA, cytosine methylation, bryophytes, epigenetics, histone marks, genome evolution

## Abstract

**Introduction:** In plants, the multicopy genes encoding ribosomal RNA (rDNA) typically exhibit heterochromatic features and high level of DNA methylation. Here, we explored rDNA methylation in early diverging land plants from Bryophyta (15 species, 14 families) and Marchantiophyta (4 species, 4 families). DNA methylation was investigated by methylation-sensitive Southern blot hybridization in all species. We also carried out whole genomic bisulfite sequencing in *Polytrichum formosum* (Polytrichaceae) and *Dicranum scoparium* (Dicranaceae) and used available model plant methyloms (*Physcomitrella patents* and *Marchantia polymorpha*) to determine rDNA unit-wide methylation patterns. Chromatin structure was analyzed using fluorescence *in situ* hybridization (FISH) and immunoprecipitation (CHIP) assays.

**Results:** In contrast to seed plants, bryophyte rDNAs were efficiently digested with methylation-sensitive enzymes indicating no or low levels of CG and CHG methylation in these loci. The rDNA methylom analyses revealed variation between species ranging from negligible (<3%, *P. formosum*, *P. patens*) to moderate (7 and 17% in *M. polymorpha* and *D. scoparium*, respectively) methylation levels. There were no differences between coding and noncoding parts of rDNA units and between gametophyte and sporophyte tissues. However, major satellite repeat and transposable elements were heavily methylated in *P. formosum* and *D. scoparium*. In *P. formosum* rDNA, the euchromatic H3K4m3 and heterochromatic H3K9m2 histone marks were nearly balanced contrasting the angiosperms data where H3K9m2 typically dominates rDNA chromatin. In moss interphase nuclei, rDNA was localized at the nucleolar periphery and its condensation level was high.

**Conclusions:** Unlike seed plants, the rRNA genes seem to escape global methylation machinery in bryophytes. Distinct epigenetic features may be related to rDNA expression and the physiology of these early diverging plants that exist in haploid state for most of their life cycles.

## Introduction

Bryophytes are the group of plants that are the closest extant relative of early terrestrial vascular plants. They comprise three separate evolutionary lineages, which are today recognized as liverworts (phylum Marchantiophyta), mosses (phylum Bryophyta), and hornworts (phylum Anthocerotophyta) (Newton et al., 2000; Cox et al., 2004; He-Nygren et al., 2006; Rensing, 2018b). In all bryophytes, the ecologically persistent, photosynthetic phase of the life cycle is the haploid gametophyte generation. Diploid sporophytes are generally very short-lived and nutritionally dependent on their gametophytes except of young capsules that are photosynthetically active (Hofmeister, 1979; Rensing, 2018a). The recently assembled genome of a moss *Physcomitrella patens* showed that the majority of its genome is composed of transposable elements from the TY3-gypsy family and other repeats (Lang et al., 2018). Its chromosomes exhibit relatively even distribution of euchromatin and heterochromatin without prominent distinction of centromeric heterochromatin. On the other hand, heterochromatic blocks have been clearly cytogenetically detected in other mosses and liverworts (Newton, 1977a; Newton, 1977b; Orzechowska et al., 2010; Orzechowska et al., 2018), suggesting variation in genomic architectures of early diverging plants.

Methylation of cytosine residues is an important epigenetic mechanism affecting vital cellular processes including genome defense and differentiation (Brautigam and Cronk, 2018; Kumar et al., 2018). In bryophytes, epigenetic mechanisms have been studied mainly in two model species, *P. patens* (moss) and *Marchantia polymorpha* (liverwort). In these genomes, similar to seed plants, the majority of methylated cytosine residues is concentrated in heterochromatic regions composed of various kind of repeats (Fukuda et al., 2004; Lang et al., 2018; Schmid et al., 2018). All three major groups of C5-DNA methyltransferases encoded by MET1, CMT2(3), and DRM2 genes were identified in the *P. patens* genome (Noy-Malka et al., 2014; Yaari et al., 2015). Consistent with these enzyme activities, the CG and CHG motifs are most frequently methylated, while the CHH motifs are less methylated. Functional studies of MET1 (Yaari et al., 2015) and CMT3 (Noy-Malka et al., 2014) mutants confirmed the essential role of DNA methylation for the *P. patens* development. Remarkable changes in genomic methylation between sporophytes and gametophytic generations have been reported in *M. polymorpha* (Schmid et al., 2018).

The ubiqutious housekeeping rRNA genes (rDNA) encode the 5S, 5.8S, 18S, and 26S ribosomal RNA (rRNA) molecules (Hemleben et al., 1988). The polycistronic 18S-5.8S-26S genes called 35S rDNA (45S rDNA in animals) are transcribed in the nucleolus organizer regions by RNA polymerase I, while the 5S rRNA genes are transcribed by RNA polymerase III in nucleoplasm. In contrast to most angiosperms where 5S rDNA occurs separate from the other rRNA genes (Garcia et al., 2017), in bryophytes, the 5S rRNA genes are linked with the 35S rDNA in all species investigated so far (Capesius, 1997; Sone et al., 1999; Wicke et al., 2011; Liu et al., 2013; Milyutina et al., 2015, Goffová et al., 2019). Similar to angiosperms, the 35S-5S rDNA repeats occupy megabase-sized regions on chromosomes comprising hundreds to thousands of units in bryophytes (Liu et al., 2013; Rosato et al., 2016). Despite high copy numbers, the intragenomic diversity is low in coding regions, at least (Liu et al., 2013; Rosato et al., 2016), indicating efficient concerted evolution (Nieto Feliner and Rossello, 2012). Cytogenetic observations indicate that the number of 35S-5S rDNA loci is also low, typically 1–2 sites per haploid chromosomal set (Nakayama et al., 2001; Orzechowska et al., 2010; Garcia et al., 2012b; Kirov et al., 2018; Orzechowska et al., 2018, Goffová et al., 2019). The rDNA units are GC-rich (Orzechowska et al., 2018) and appear to be highly condensed in interphase (Rosato et al., 2016; Orzechowska et al., 2018).

A large body of data have been accumulated about the dense methylation of rDNA loci (both 35S and 5S) analyzed by means of conventional methylation-sensitive restriction endonuclases (Goldsbrough et al., 1981; Gottlob-mchugh et al., 1990; Komarova et al., 2004) and bisulfite sequencing (Fulnecek et al., 1998; Lawrence et al., 2004; Simon et al., 2018) in seed plants, while no or little information exists about methylation landscapes of rDNA in early diverging plants. The subtelomeric positions that frequently accumulated rDNA repeats (Garcia et al., 2017) were shown to be heavily methylated in *Marchantia paleacea* (Takio et al., 1999). Recently, a contig containing several rDNA units derived from the major *P. patens* rDNA locus was reported to be largely euchromatic without significant cytosine methylation and heterochromatic marks, suggesting that it derived from an active part of the array (Kirov et al., 2018). We aimed to fill a gap in our knowledge of bryophyte epigenetic landscape by focusing on methylation of their rDNA. Using both conventional restriction mapping and whole genomic approaches, we determined methylation levels of 35S and 5S rDNA in a wide range of Bryophyta and Marchantiophyta genera and compared the data with those of seed plants. In addition, we analyzed epigenetic histone marks along the rDNA units in selected species by chromatin immunoprecipitation.

## Materials and Methods

### Plant Material

Living specimens belonging to 19 bryophyte species were collected from Czechia, Continental Spain, Balearic Islands, and Slovenia (**Table 1**). Sampling was carried out during the years 2017–2019 (Czech Republic) and year 2012 (other countries, Rosato et al. 2014). For geographical localities of accessions, see **Supplementary Table S1**. *P. patens* was vegetatively propagated in a lab following standard procedures (Hola et al., 2013). DNA samples of *Tragopogon mirus*, *Nicotiana tabacum*, *Iris versicolor*, and *Ginkgo biloba* were from our previous works (Dobesova et al., 2015; Wang et al., 2016).

**Table 1:** List of species and type of analyses.

Plant division	Species	Family	Type of analysis^1^
Bryophyta	*Bryum capillare* Hedw.	Bryaceae	SB
	*Dichranum scoparium* Hedw.	Dicranaceae	SB,HTP
	*Calliergonella cuspidata* (Hedw.) Loeske	Hypnaceae	SB
	*Encalypta streptocarpa* Hedw.	Encalyptaceae	SB
	*Fissidens dubius* P. Beauv.	Fissidentaceae	SB
	*Hypnum cupressiforme* Hedw.	Hypnaceae	SB
	*Funaria hygrometrica* Hedw.	Funariaceae	SB
	*Physcomitrella patens* (Hedw.) B.S.G. ^2^	Funariaceae	HTP, SB
	*Plagiomnium undulatum* (Hedw.) T.J. Kop.	Miniaceae	SB
	*Polytrichum formosum* Hedw.	Polytrichaceae	CHIP, FISH, HTP, MNase, SB,
	*Pleuzorium schreberii* Hedw.	Hylocomiaceae	SB
	*Schistidium apocarpum* (Hedw.) Bruch & Schimp.	Grimmiaceae	SB
	*Sphagnum denticulatum* Brid.	Sphagnaceae	SB
	*Syntrichia ruralis* (Hedw.) F. Weber & D. Mohr ^3^	Pottiaceae	SB
	*Thamnobryum alopecurum* (Hedw.) Gangulee	Neckeraceae	SB
Marchantiophyta	*Bazzania trilobata* (L.) Gray	Lepidoziaceae	SB
	*Conocephalum conicum* (L.) Underw.	Conocephalaceae	SB
	*Marchantia polymorpha* L. subsp. *polymorpha*	Marchantiaceae	SB, HTP
	*Porella platyphylla* (L.) Pfeiff.	Porellaceae	SB
Magnoliophyta	*Arabidopsis thaliana*(L.) Heynh.	Brassicaceae	HTP
	*Brassica napus* L.	Brassicaceae	HTP
	*Iris versicolor* L.	Iridaceae	SB
	*Nicotiana tabacum* L.	Solanaceae	SB
	*Oryza sativa* L.	Poaceae	HTP
	*Solanum lycopersicum* (L.) 1753	Solanaceae	HTP
	*Theobroma cacao* (L.) 1753	Malvaceae	HTP
	*Tragopogon mirus* Ownbey	Asteraceae	SB
Ginkgophyta	*Ginkgo biloba* L.	Ginkgoaceae	SB

^1^SB—Southern blot hybridization, HTP—high-throughput bisulfite sequencing, CHIP—chromatin immunoprecipitation, FISH—Fluorescence in situ hybridization, MNase—chromatin nuclease sensitivity assay. 2Strain “Gransden 2004.” 3Var. ruralis. For a geographical location of plant species collected within this study, see **Supplementary Table S1**.

### DNA Isolation

Gametyphyte DNA was isolated from green parts of plants in the laboratories of University of Valencia and Czech Academy of Sciences in Brno. Usually, 5–10 shoots (approximately 0.5 g) were used for DNA extraction. Sporophyte DNA was isolated from brownish seta (without the capsules) collected from about 100 P. *formosum* individuals in the middle of June 2018. Genomic DNA was extracted using the modified cetyl trimethylammonium bromide method (Kovarik et al., 1997). In some cases, plants were lyophylized prior to DNA extractions to decrease the water content. The quality of DNA was evaluated by agarose gel electrophoresis and quantity assessed by a fluorometer (Quantus, Promega, USA).

### High-Throughput Sequencing

About 3–5 μg of high molecular weight (>20 kb) genomic DNA from *P. formosum* and *D. scoparium* (native and bisulfite-treated) was sequenced at the BGI (Hong Kong) Company. The source plants originated from populations in a suburban area of Brno, Palackeho vrch, Czech Republic (WGS-84 coordinates: 49.2255433N, 16.5686750E). Genome coverage, read quality, and other parameters are in **Supplementary Table S2**.

### Assembly of rDNA Units

The rDNA units were assembled from *P. formosum* and *D. scoparium* genomic reads according to the procedures described in Wang et al. (2016). Briefly, 200,000 pair end reads from each genome were subjected to clustering analysis using RepeatExplorer pipeline (Novak et al., 2013). Clusters containing rDNA sequences were selected and the cluster contigs exported to the CLC Bio Genomics Workbench (CLC). The order of genes in contigs was checked by BLASTing sequences against the 18S rDNA sequences from *P. formosum* (GenBank X80982; Capesius and Stech, 1997) and *M. polymorpha* (AB021684; Sone et al., 1999). Additional GenBank clones were used during assembly: *P. formosum* ITS2 (AY396435; Hyvonen et al., 2004), *Funaria hygrometrica* 35S rDNA unit (X80212; Capesius, 1997), and *D. scoparium* 18S rDNA (X89874; Capesius and Stech, 1997). rDNA units were assembled using a tool “Assemble Sequences” in the CLC, and the contigs were aligned with the GenBank clones. Consensus sequences were generated from each alignment. The short contigs with short overlaps were added to the long contigs using a command “Add sequences to contig.” The assembled sequences were checked for correct order by alignment with 18S and 26S rDNA sequences, annotated and used as a reference sequences in further analyses.

The copy number of rDNA units was calculated from the Illumina read count using the following procedure: 1) Genome proportion was calculated from the number of mapped reads to the 18S and 5S rDNA reference divided by the total reads and expressed in percentage. 2) Calculation of genome space: genome proportion × size of the genome in Mb. 3) Copy number was then calculated: genome space values in Mb divided by the size of the unit.

For single nucleotide polymorphisms (SNPs) calling, we used mapped reads as source files. The reference sequences were 18S-5.8S-26S-5S units [without intergenic spacer (IGS) subrepeats] obtained from assembled sequences. Mapping parameters: 0.5_Length fraction, 0.8_Similarity fraction. 3_Insertion cost, 3_Deletion cost. SNPs were called using a “Basic variant function” of CLC using the following parameters: 200_Minimum coverage, 20_Minimum counts, 10_Minimum frequency.

### Ribosomal DNA Methylation Analysis From Whole Genomic Bisulfite Sequencing

Whole genomic bisulfite DNA reads (Illumina) comprised four bryophyte (*D. scoparium*, *M. polymorpha*, *P. formosum*, and *P. patens*) and five angiosperm (*Arabidopsis thaliana*, *Brassica napus*, *Oryza sativa*, *Solanum lycopersicum*, and *Theobroma cacao*) species. Leaf DNAs from *D. scoparium* and *P. formosum* were *de novo* sequenced in this work; the remaining sequence reads archives were obtained from public databases (**Supplementary Table S3**). Reads were quality-checked with CLC genomics tools filtered to uniform lengths (100–150 bp) and quality (Phred scores ≥30). Pair end or single end reads were mapped (CLC genomic, epigenomic tool) to the 18S rDNA and 5S rDNA reference sequences specific to each genome or to the whole 35S rDNA contigs obtained and described previously. Mapping parameters were as follows: 0.8_Length fraction, 0.8_Similarity fraction, and a nondirectional option was selected. Poorly covered or exceptionally highly covered region derived from repetitive regions were excluded from the analysis. Reads were mapped to two reference sequences derived from the initial native sequence in which all Cs were replaced by Ts (revealing cytosine methylation in the upper DNA strand) or from Gs into Cs (methylation analysis of the bottom DNA strand). Reads or matches mapped ambiguously were removed. Call methylation levels parameters were as follows: duplicate matches were allowed. The program generated two files including a tabular summary overview of rDNA-wide methylation levels for CG, CHG, and CHH contexts and a track displaying information about the position, strand, context, and methylated coverages. Methylation levels were calculated by dividing “Methylation coverage” values with “Context coverage” values.

### DNA Probes

For Southern blots, the 26S probe was a 220-bp fragment obtained by amplification of a 26S rRNA gene from *P. formosum* according to a protocol described in Lim et al. (2000). The noncoding PforCL1 repeat of *P. formosum* was amplified based on the cluster contig using forward (5’-ATTGGTGGAAAAGGCGG-3’) and reverse (5’AGAGCCCAAAAGAAAGTGT-3’) primers. A single 398-bp fragment was obtained and labeled as discussed later. For FISH, the 5S probe was an insert of the clone containing three copies of a 5S rRNA gene from *Artemisia tridentata* (Garcia et al., 2012a); the 18S rDNA probe was an insert of the plasmid carrying a tomato 18S rRNA gene (Kiss et al., 1989).

### Southern Blot Hybridization

Purified genomic DNAs (∼2 μg/sample) were digested with the restriction enzymes *Msp*I, *Hpa*II, *Bst*NI, or *PspG*I and separated by gel electrophoresis on a 0.9% (w/v) agarose gel. The gels were then alkali-blotted onto Hybond-XL membranes (GE Healthcare, Little Chalfont, UK) and hybridized with a ^32^P-labeled DNA probe (DekaLabel kit, Thermofisher Scientific, USA) for the 26S and 18S ribosomal RNA (rRNA) genes and PforCL1 repeat. Hybridization followed protocols described in Kovarik et al. (2005). After washing (2 × 5 min with 2× SSC, 0.1% sodium dodecyl sulfate at room temperature followed by 2 × 15 min with 0.2× SSC, 0.1% sodium dodecyl sulfate, 65°C), the hybridization bands were visualized with a PhosphorImager (Typhoon 9410, GE Healthcare, PA, USA) and the data quantified by ImageQuant software (GE Healthcare, PA, USA). Completeness of restriction enzyme digestions was checked by re-hybridization of blots with a chloroplast rbcL gene probe from tobacco - chloroplast DNA is known to lack cytosine methylation (Fojtova et al., 2001).

### Chromatin Immunoprecipitation

About 1.5 g of *P. formosum and T. mirus* leaf was extensively washed in distilled water, dried in filter paper layers, and incubated with 1% formaldehyde (92.5 ml) under vacuum for 10 min. Crosslinking reaction was terminated by addition of 5.5 ml of 2-M glycine, and vacuum was applied for another 5 min. Fixed material was extensively washed by water, dried and kept frozen at −70°C until use.

**Figure 1:**
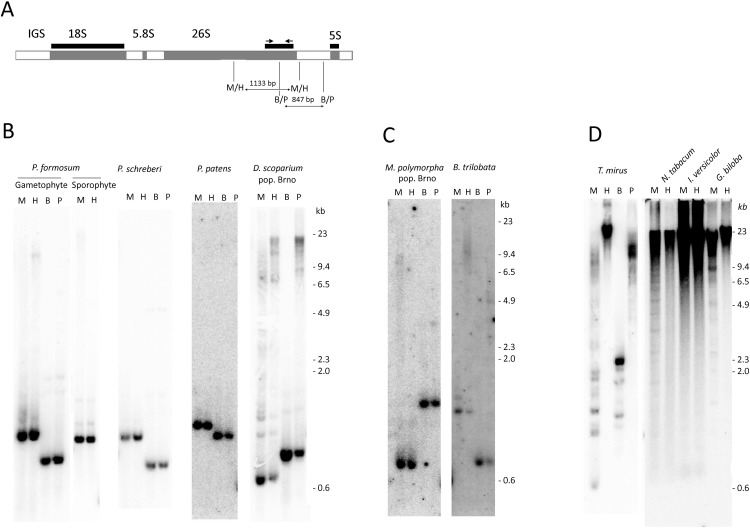
Analysis of rDNA methylation by methylation-sensitive restriction enzymes. **(A)** Scheme of rDNA unit showing positions of hybridization probes and PCR primers. **(B**–**D)**. Southern blot hybridization of the 26S rDNA probe to genomic DNAs digested with *Msp*I/*Hpa*II and *Bst*NI/*Psp*GI isoschizomeres. **(B)** Mosses, **(C)** Liverworts, **(D)** Seed plants. Lane labels: H—*Hpa*II, M—*Msp*I, B—*Bst*NI, P—*Psp*GI restriction enzymes.

The EpiQuik^™^ Plant ChIP Kit (Epigentek, New York, USA) was used for chromatin immunoprecipitation. The protocol followed the manufacturer’s recommendations with the following modifications. Protease inhibitors were added to incubation buffers: The CP3D buffer was supplemented with 7.5 μg/ml pepstatin and 150-μM phenylmethylsulfonyl fluoride (PMSF); the CP3C buffer was supplemented with 8.3 μg/ml of each leupeptin and pepstatin and 167-μM PMSF. DNA was eluted with 2 × 8 μl elution buffer for 1 min each and stored at –20°C until use. Immunoprecipitation was performed using commercially available antibodies, such as anti-H3K4me3 (Abcam; catalog no. AB8580), anti-H3K9me2 (Abcam, catalog no. AB1220), and anti-H3 (Abcam catalog no. AB1791), recognizing total histone H3 independently of whether it is modified or not. Real-time polymerase chain reaction (PCR) was performed on ChIP samples in a Rotor-Gene 6000 Series thermocycler. The 26S rRNA gene (3’ coding region) was amplified with the forward primer 5’-GAATTCACCCAAGTGTTGGGAT-3’ and the reverse primer 5´-AGAGGCGTTCAGTCATAATC-3´ (positions of primers are depicted in **Figure 1A**). The 20-μl PCR reaction mixture consists of 1-μl immunoprecitated DNA, 10-μl 2× FastStart SYBR Green Master Mix (Roche), 1 μl of each primer (10 µM), and 8-μl distilled water. The reaction conditions were as follows: Initial denaturation at 92°C for 10 min followed by 45 cycles of 92°C, 20 s; 57°C, 20 s; 72°C, 30 s. PCR reactions were performed in triplicate. Quality of products was checked by gel electrophoresis. A single band of the expected size (220-bp) was produced. The biological replicate included material from three independent crosslinking reactions of plants from the same population.

### Micrococcal Nuclease Digestion of Chromatin

Nuclei of *P. formosum* were prepared for micrococcal nuclease (MNase) treatments using a protocol modified from Espinas and Carballo (1993). All operations were performed at 0–4°C. Approximately 1.5 g of fresh plants was grounded in liquid nitrogen and resuspended in 50 ml of buffer A [10-mM sodium chloride (NaCl), 10-mM 2-ethanesulfonic acid pH 6.0, 5-mM ethylenediaminetetraacetic acid (EDTA), 0.15- mM spermidine, 20-mM β-mercaptoethanol, 0.6% TRITON X-100, 0.2-M sucrose, and 0.1-mM PMSF]. The homogenate was filtered through nylon mesh (109 µm) and centrifuged at 2,620×*g* for 10 min. The pellet was resuspended in 15 ml of buffer A, centrifuged again, resuspended in 15 ml of buffer B (88% w/w Percoll in buffer A), and centrifuged at 5,150×*g* for 10 min. Floating nuclei (0.4 ml) were resuspended in 1 ml of MNase digestion buffer (50-mM Tris-HCl pH 8.0, 125-mM sucrose, 75-µM spermine, 5-mM magnesium chloride, 3-mM calcium chloride, 250-µM spermidine, 0.1-mM PMSF, and 20 mM β-mercaptoethanol) and treated with 30 units/ml of MNase at 37°C. Aliquots were removed from the reaction mixture at various times and the digestion stopped with equal volumes of stop buffer (1% sarcosyl, 0.25-M EDTA pH 8, 5-mM egtazic acid, and 0.5-M NaCl). The samples were treated with proteinase K (several hours at 52°C), extracted with phenol–chloroform–isoamyl alcohol and ethanol precipitated. Five micrograms of DNA was fractionated on a 2% agarose gel and hybridized to rDNA or the satellite repeat.

### Isolation of Nuclei and Fluorescence *in Situ* Hybridization

*Polytrichum formosum* leaves were placed in Tris-HCl buffer (1 M, pH 7.5) before isolation. A new Petri dish with 300 μl of LB1 buffer [15-mM Tris-HCl pH 7.5, 2-mM EDTA, disodium salt dihydrate, 0.2-mM spermine, 80-mM potassium chloride, 20-mM NaCl, 15-mM β-mercaptoethanol, and 0.1% (v/v) Triton X-100] (Dolezel et al., 1989) was placed on ice and small amount of leaves (5-7) was chopped to very small pieces using a razor blade. The mixture was filtered through the 30-nm nylon mesh. However, for *D. scoparium*, this protocol did not provide satisfactory results. We therefore isolated *D. scoparium* nuclei by homogenization of tissue in a liquid nitrogen followed by a Percoll gradient as described in a previous section. About 30 μl of nuclei solution was pipetted directly on the prechilled slides. It was important to remove the slides from the dry ice to avoid freezing of the buffer and apply the nuclei solution 2 or 3 s afterward. The slides were then incubated in the Carnoy’s fixative for 20 min. When air dried, the slides were used for FISH. FISH followed the procedures described in Herklotz et al. (2018). The probes were labeled by nick translation using Spectrum green deoxyuridine triphosphates (Abbott, USA) for 18S rDNA and Cy3- deoxyuridine triphosphates (Roche, Switzerland) for 5S rDNA. The slides were scanned using epifluorescence microscopes Olympus Provis AX70 with ISIS imaging software (MetaSystems, Germany). The Adobe Photoshop version 11.0 software was used to optimize the images.

## Results

### Ribosomal DNA Analysis Using Methylation-Sensitive Restriction Enzymes

We employed the methylation-sensitive restriction assay that measured DNA methylation within restriction sites in a population of many rDNA molecules. Both *Msp*I and *Hpa*II enzymes recognize the same CCGG target sequence; however, *Msp*I is unable to digest when the first cytosine is methylated, making it sensitive to mCCG methylation. *HpaII* is unable to cleave the site when the second cytosine is methylated, making it sensitive to CG methylation. **Figure 1A** depicts regions of probe hybridization and the positions of restriction sites within the *P. formosum* 35S-5S rDNA unit. The *Msp*I and *Hpa*II restriction enzymes produced identical low molecular hybridization bands in all Bryophyta and Marchantiophyta species (**Figures 1B, C** and **Supplementary Figures S1A, C**). There were no significant differences in hybridization profiles between two populations of each *D. scoparium* and *M. polymorpha*. In contrast, DNA from representative seed plant species showed mostly high molecular weight hybridization signals (**Figure 1D**) consistent with a dense methylation pattern of both external and internal Cs in the CCGG recognition sites. DNA from diploid seta (sporophyte) of *P. formosum* showed similar hybridization profiles as the DNA from the haploid leaf tissue (gametophyte) (**Figure 1B**). In order to analyze methylation in CWG contexts, genomic DNAs were digested with the *Bst*NI and *Psp*GI isoschizomeres both cutting at CCWGG motifs. While *Psp*GI is sensitive to methylation of the second C, *Bst*NI is insensitive to C-methylation. In bryophytes, both *Bst*NI and *Psp*GI produced similar 1–2 low (<3 kb) molecular weight fragments after the hybridization with the 26S rDNA probe (**Figure 1** and **Supplementary Figure S1C**), and only *D. scoparium* (**Figure 1B**) showed a minor *Psp*GI-resistant fraction. In contrast, rDNA from *Tragopogon mirus* (angiosperm species) was remarkably resistant to the *Psp*GI digestion (**Figure 1D**) indicating a high level of CWG methylation in this species.

### Assembly and Intragenomic Homogeneity of rDNA Units

Using high-throughput genomic data, we assembled 35S and 5S rDNA units in *P. formosum* and *D. scoparium*. In both species, the contigs showed a conserved structure encoding the 18S-5.8S-26S-5S (in this order) genes (**Supplementary Figure S2**). Coordinates and lengths of individual subregions of assembled units are given in **Supplementary Table S4**. The 5S rRNA located downstream from the 26S gene was encoded by the same DNA strand as the 26S rRNA (direct orientation). The first 5S codon was preceded by a GTCG motif that appeared to be conserved in the distantly related *Schistidum* (Grimmiaceae) IGS1 (Milyutina et al., 2015). The TATA box was found in *P. formosum* but not in *D. scoparium* sequence. The intergenic spacer (IGS1) between 26S rDNA and 5S rDNA was generally non-repetitive and was not conserved between both species. BLAST searches did not reveal significant similarity to IGS1 from other liverwort (*Marchantia*) and moss genera (*Schistidium*, *Funaria*). The IGS1 length (approximately 400 bp) was comparable between both species. The IGS2 downstream from the 5S rDNA insertion was not fully assembled probably because of its highly repetitive structure (**Supplementary Figure S3B**). The lengths of the internally transcribed spacers were not conserved across species. *P. formosum* had ITS1 longer than ITS2 contrast to *D. scoparium* in which ITS2 was longer than ITS1. Overall, there were no significant subrepeats in ITS1 and ITS2.

The genomic Illumina reads were mapped to the 18S-5.8S-26S regions (without ETS and IGS) allowing comparison of genetic variation between the species. The homogeneity of rDNA units was high with no variation in genic sequences and both ITS regions (**Supplementary Figure S2**). The copy number of 18S and 5S rDNA was calculated from high-throughput data (**Supplementary Table S5**). In bryophytes, the rDNA copy number range (500–2,600 per haploid genome) was consistent with a previous report (Rosato et al., 2016).

### High-Resolution Cytosine Methylation Analysis of rDNA by Whole Genomic Bisulfite Sequencing

**Figure 2:**
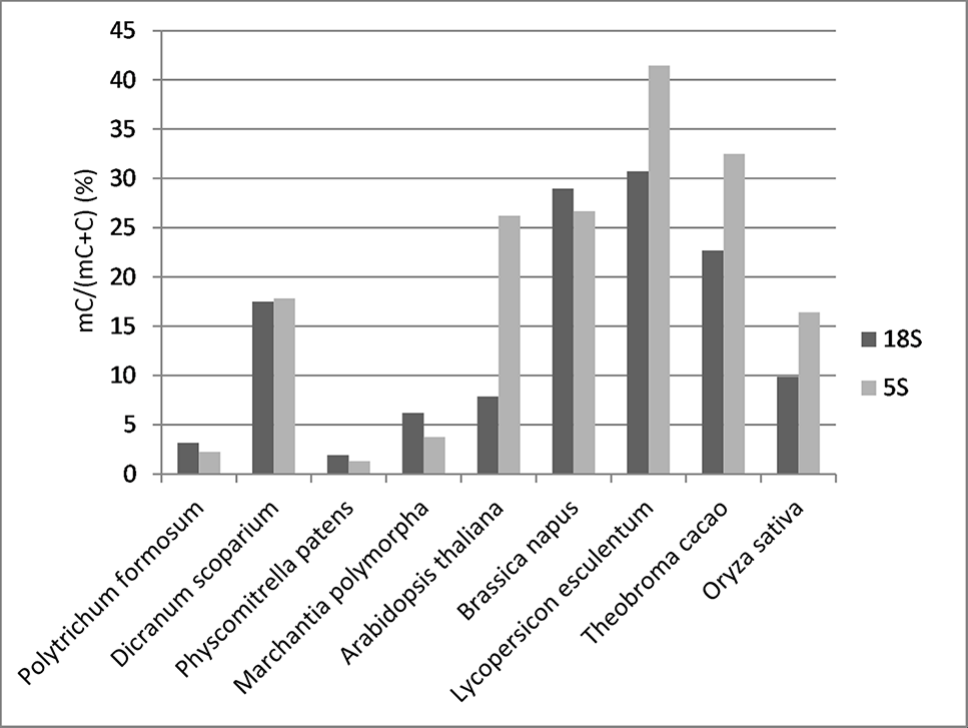
Overall cytosine methylation levels in 18S and 5S rRNA genes established by bisulfite sequencing.

To determine the distribution of cytosine methylation across rDNA units, we sequenced bisulfite-treated genomic DNA from *P. formosum* and *D. scoparium*. We also used available bisulfite read archives from other projects including *P. patens*, *M. polymorpha*, *Arabidopsis thaliana*, *Brassica napus*, *Oryza sativa*, *Lycopersicum lycopersicum*, and *Theobroma cacao* (IDs of SRA archives are given in **Supplementary Table S3**). We first analyzed a global methylation in 18S rDNA and 5S rDNA. Mapped bisulfite provided smooth and even read coverages along the approximately 1,800-bp (18S rRNA gene) and 120-bp (5S rRNA gene) reference sequences. Comparison of methylation levels between divergent groups of species is shown in **Figure 2**. Both *P. formosum* and *P. patens* contained almost no methylation in their rDNAs, while *M. polymorpha* and, particularly, *D. scoparium* showed methylation in both rRNA genes.

**Figure 3:**
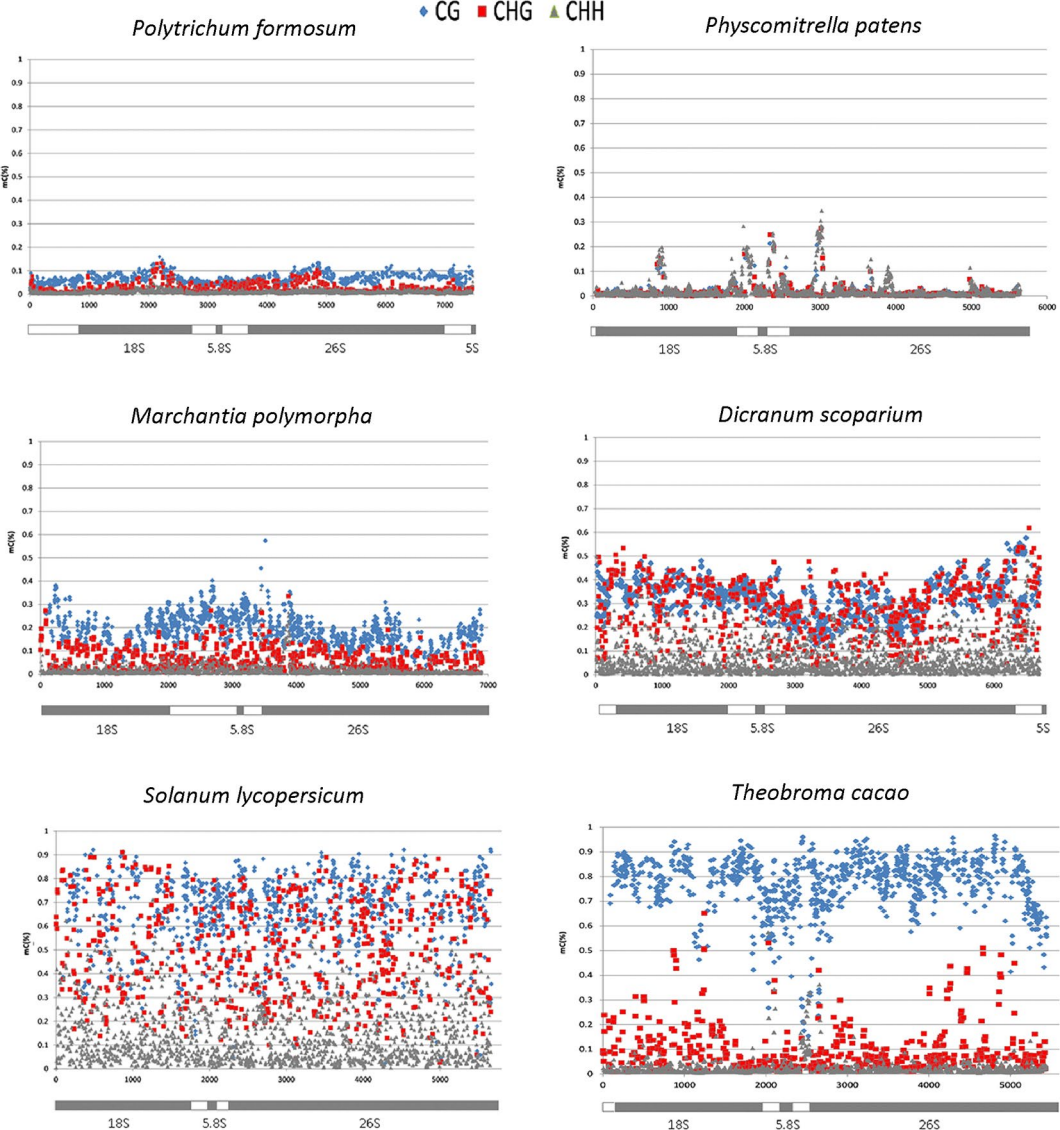
Distribution of cytosine methylation along the rDNA units in four bryophyte and two angiosperm species. The diagrams below the charts correspond to rDNA regions that have been analyzed at a single C resolution. Each symbol represents the methylation level calculated for each C within the CG (blue), CHG (red), and CHH (gray) motifs (y-axis, 1 = 100% methylation).

In four bryophyte and two seed plant species, we analyzed the distribution of methylation along the whole rDNA units (rDNA methylom) including both ITSs and non-repetitive parts of IGS subregions (**Table 2**). In each DNA strand, about 2,000 cytosine residues were evaluated. Proportions of mCs were plotted against the C positions (**Figure 3**). Both *P. formosum* (A) and *P. patens* (B) showed almost the absence of cytosine methylation in all sequence contexts. *D. scoparium* had considerable methylation of rDNA, while its level was lower than any of the angiosperm species. The CG methylation was high in both *T. cacao* and *S. lycopersicum*. However, *T. cacao* had relatively low level of CHG methylation compared with *S. lycopersicum*. In all species, the cytosine methylation was relatively evenly distributed along the units. Mapping of reads to the IGS subrepeats located downstream of the 5S rRNA genes resulted in artifacts evidenced by unrealistically high methylation calls (**Supplementary Figure S3A**). Haplotypic analysis in the *D. scoparium* 18S rDNA subregion (**Supplementary Figure S4**) revealed that methylation was distributed asymmetrically, i.e., some units were densely methylated, while the others were completely unmethylated, while intermediately methylated molecules were rare.

**Table 2:** Methylation levels in rDNA units and noncoding repeats determined from high-throughput bisulfite sequencing.

Species	Repeat element type^1^	Genome abundance	Methylation level [%]			
		[%]	CG	CHG	CHH	total
D. scoparium	35S-5S rDNA^2^	1.05	31.3	26.1	6.0	16.6
	Retroelement/Ty3_copia	2.22	75.3	63.6	24.2	43.1
	satellite	0.14	nd^4^	84	14.4	17.9
	featureless repeat 1	0.48	68.3	55.8	28.1	36.9
	featureless repeat 2	0.38	64.1	36.5	19.8	35.7
P. formosum	35S-5S rDNA^2^	0.78	6.7	2.8	1.1	2.4
	Retroelement/Ty3_gypsy	0.24	76.8	20.6	4.0	15.6
	ClassI/Line-RT	0.29	66.5	28.9	11.8	17.5
	satellite	0.08	90.4	41.4	8.3	22.8
	featureless repeat	0.42	51.4	9.8	2.3	22.2
M. polymorpha	35S rDNA^3^	1.64	18.0	6.4	1.5	6.9
P. patens	35S rDNA^3^	0.9	1.8	1.7	1.4	1.7
S. lycopersicum	35S rDNA^3^	1.66	60.1	46.4	15.6	33.0
T. cacao	35S rDNA^3^	0.31	73.5	9.3	4.0	24.2

^1^Data are from the RepeatExplorer cluster analysis of whole genomic DNA reads.

^2^Cytosine methylation was estimated in 35S-5S units without IGS2.

^3^Cytosine methylation was estimated in the 35S region without 5S genes.

^4^There were no CG motifs in the satellite monomer consensus.

Using the RE pipeline, we recovered consensus sequences of several noncoding repeats in each *P. formosum* and *D. scoparium* and determined their methylation status (**Table 2**). In general, methylation of the repeats in both species was highly independent of their origin. In *P. formosum*, the methylation status of CL1 repeat (PforCL1) was verified by Southern blot hybridization (**Supplementary Figure S1B**).

### Chromatin Nuclease Sensitivity Assays Reveals Canonical Nucleosomal Structure of Bryophyte rDNA

**Figure 4:**
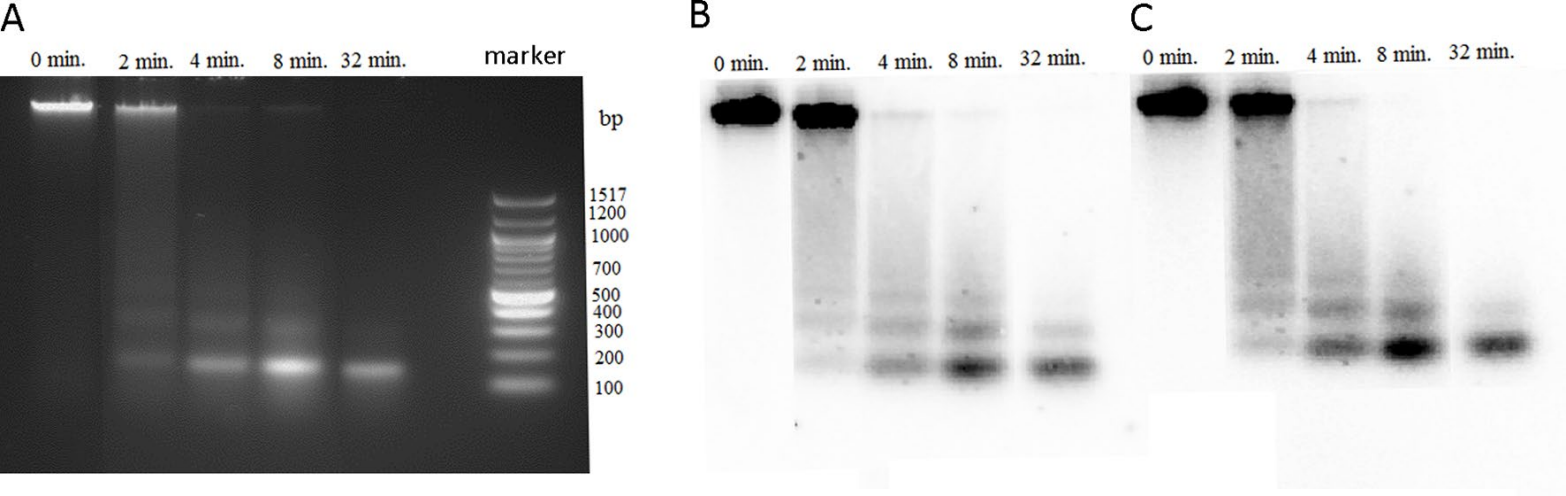
Accessibility of *P. formosum* chromatin to micrococcal nuclease (MNase). Agarose gel electrophoresis of the MNase digestions products from different time point intervals. **(A)** Ethidium bromide-stained gel before blot. Blot hybridized with the 26S rDNA **(B)** and PformCL1 **(C)** probes.

Highly methylated regions of genome usually display resistance to the MNase digestion compared with euchromatic less methylated regions. It was of interest to determine the MNase accessibility of rDNA chromatin in *P. formosum* that has negligible methylation of its rDNA. We digested the *P. formosum* chromatin with MNase at different time point intervals, and DNA from each interval was analyzed through agarose gel electrophoresis followed by Southern blot hybridization with the 26S rDNA and PfoCL1 probes (**Figure 4**). The MNase digestion profiles show similar nucleosomal fragmentation of bulk (left panel), 26S rDNA (middle panel), and PfoCL1 (right panel) chromatins. Thus, most rDNA is packed in a canonical nucleosomal structure with a repeat periodicity of about 180 bp. The kinetics of MNase digestibility was similar between bulk chromatin and both rDNA and PforCL1 loci.

### Balanced Euchromatic and Heterochromatic Histone Marks in *P. formosum* rDNA

**Figure 5:**
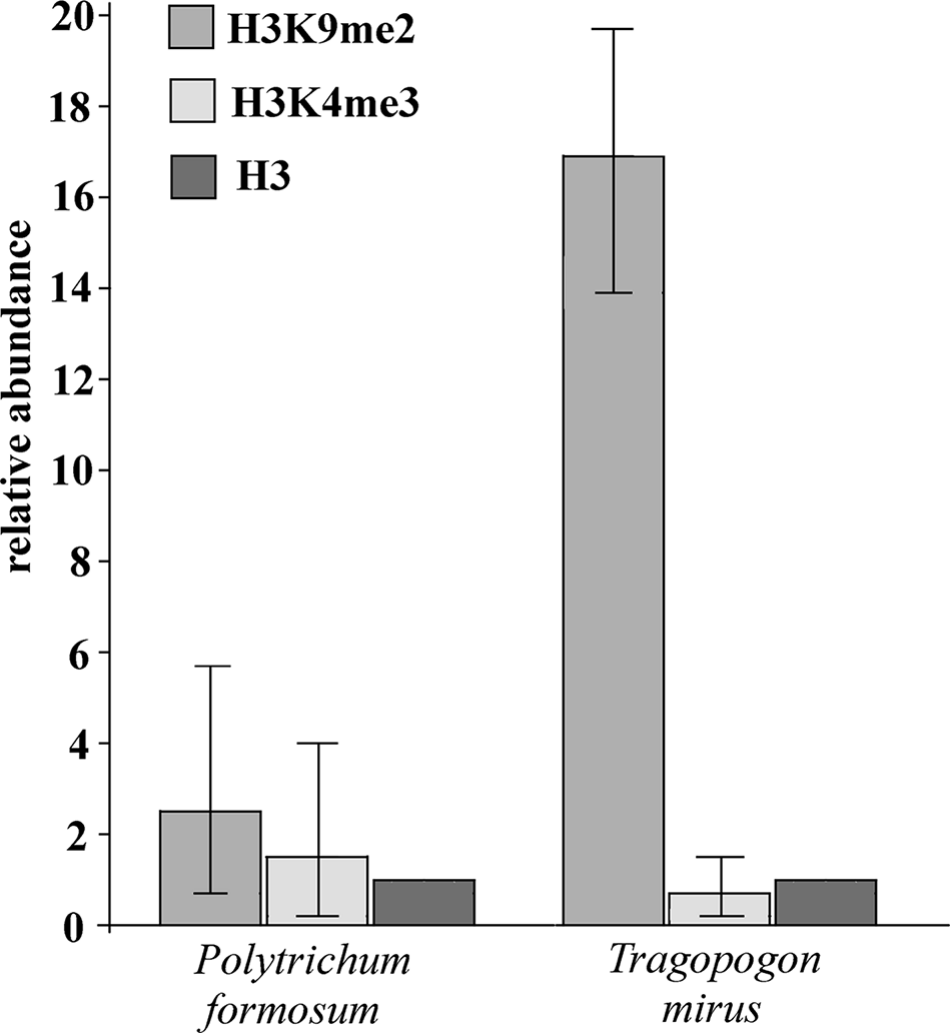
Relative content of heterochromatic and euchromatic histone H3 epigenetic marks in rDNA chromatin. The enrichment of immunoprecipitated H3K9m2 and H3K4m3 relative to unmodified histone H3 is shown for *P. formosum* moss and *T. mirus* angiosperm (data were averaged from the three biological replicates, **Supplementary Table S6**). Y-axis-fold enrichment over the H3 signal.

We examined histone modification marks in the 26S rRNA gene in moss *P. formosum* and *T. mirus* (angiosperm). Chromatin isolated from purified nuclei was immunoprecipitated with antibodies against H3K4m3 (euchromatic mark) and H3K9m2 (heterochromatic mark) and unmodified histone H3. The enrichment of individual epigenetic marks relative to histone H3 is shown in **Figure 5**. It is evident that the heterochromatic H3K9m2 mark was only slightly (twofold) more abundant than the euchromatic H3K4m3 mark in *P. formosum*. In contrast, the heterochromatic H3K9m2 was highly (33-fold) enriched in the *T. mirus* rDNA chromatin compared with the euchromatic H3H4m3 mark.

### Fluorescence *in Situ* Hybridization Reveals High Level of rDNA Chromatin Condensation in Interphase

**Figure 6:**
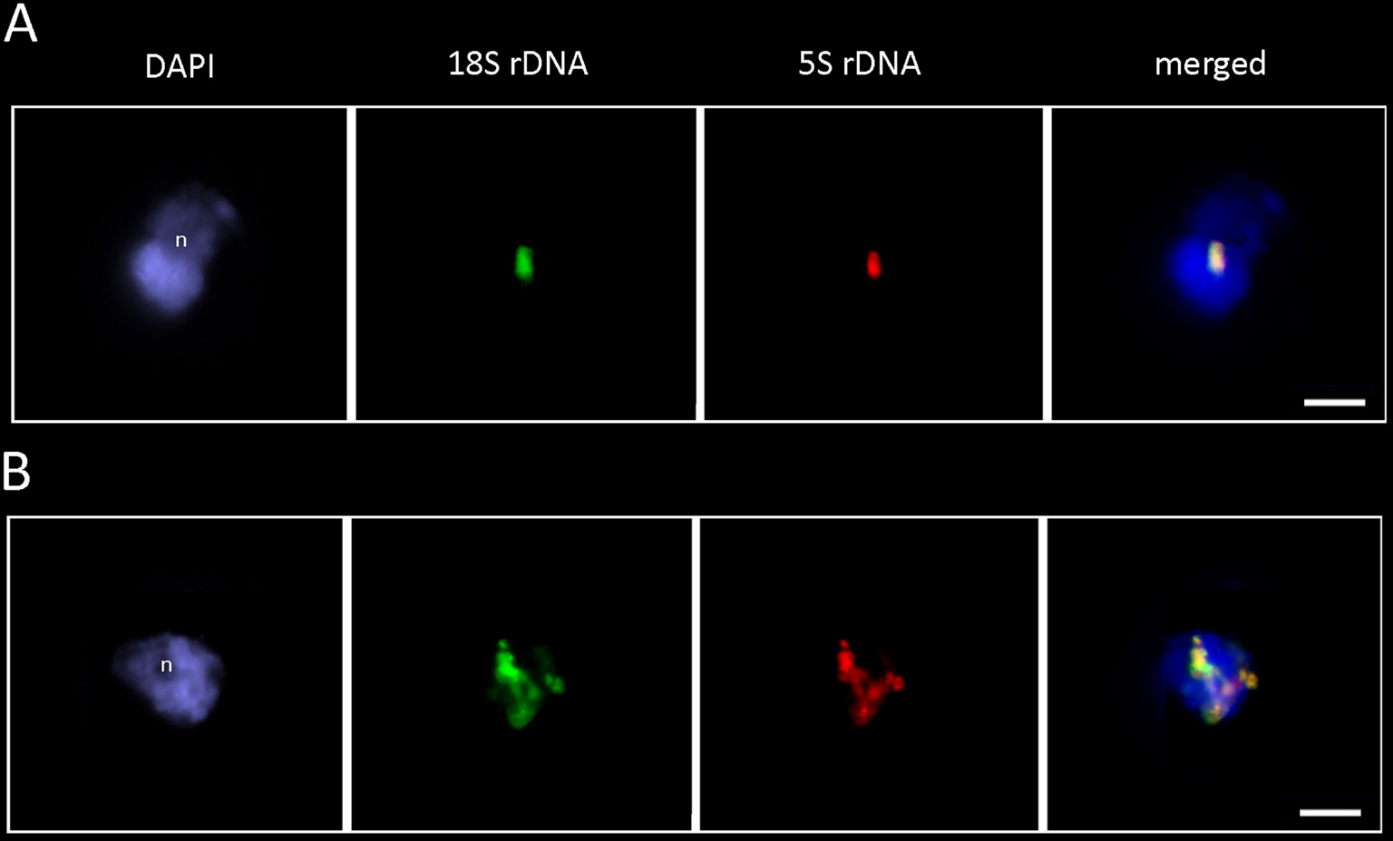
FISH analysis of *P. formosum* **(A)** and *D. scoparium* **(B)** rDNA. The interphase nuclei hybridized with the 18S rDNA (green) and 5S rDNA (red) probes. Note, highly condensed rDNA signals at the nucleolar periphery. Note, overlapping signals of 18S and 5S rDNA probes. n—nucleolus. Scale bar—10 µm.

To determine the structure of rDNA chromatin in interphase, we hybridized the *P. formosum* and *D. scoparium* nuclei with the 18S and 5S rDNA probes (**Figure 6**). In both species, the 18S rDNA (green signal) and 5S rDNA (red signal) probes colocalized, confirming linkage of both genes. The fluorescent signals were highly condensed and localized mostly at the nucleolar periphery. In addition to condensed clusters, *D. scoparium* showed partially decondensed signals stretching across the nucleus indicating transcription activity of rDNA units.

## Discussion

Ribosomal RNA genes represent a classical heterochromatic locus displaying high levels of cytosine methylation in seed plants and most animals. Surprisingly, a wide range of bryophytes from diverging taxonomic groups that we analyzed here show little or no methylation in both 35S and 5S loci. The rDNAs were unmethylated both in gametophyte and sporophyte tissues suggesting that, within species, the ploidy level and developmental stage do not influence methylation (its absence) at this locus. These results are interesting, since it has been reported in *Marchantia* species that the appearance of sexual structures in the gametophyte is linked to the expression of new polyphenol compounds (Markham et al., 1978). Moreover, in *F. hygrometrica*, a flavonoid-like compound (bracteatin) is produced in the sporophyte (capsule), while it is absent in the gametophyte (Weitz and Ikan, 1977). Although the genetic basis of such changes has not been addressed, it is likely that epigenetic changes related to gene methylation are involved. The methyl cytosine density was relatively uniform across the rDNA units with no apparent differences between coding and noncoding regions. This is in line with a comparative whole genomic methylation analysis of *M. polymorpha* that showed reduced 35S rDNA methylation compared with other repeats (Schmid et al., 2018). In *P. patens*, a contig carrying several rDNA copies seemed to be almost devoid of DNA methylation (Kirov et al., 2018). The authors suggested that the contig may derive from an active part of the array and that inactive condensed rDNA arrays may have different methylation patterns. However, our results indicate that in *Physcomitrella*, whole rDNA arrays are likely to be non-methylated or severely undermethylated without major methylation heterogeneity. The key methylation enzymes are well conserved in bryophytes (Noy-Malka et al., 2014; Yaari et al., 2015) and are likely involved in global methylation of bryophyte genomes. Indeed, several noncoding high copy repeats that we analyzed here were heavily methylated in *P. formosum* and *D. scoparium*. However, the data presented in this work indicate that rDNA loci escape global methylation machinery in these early diverging plants.

### Variation in rDNA Methylation Between Bryophyte and Seed Plant Species

The differences between seed plants and bryophytes in rDNA methylation are striking. Why there is so little methylation in bryophyte rDNA? Several hypotheses could be drawn:

- rDNA methylation may be influenced by gene copies. In wheat, a correlation between rDNA copy number and rDNA methylation was observed (Sardana et al., 1993). In line with this supposition, a positive correlation between copy number and degree of methylation was observed (R^2^ = 0.804, P < 0.005, **Supplementary Figure S5**) when both seed and non-seed species were included. However, notable exceptions from this trend exist. For example, *M. polymorpha* has twice as many copies (approximately 2,600 copies) compared with *D. scoparium* (approximately 1,000) despite its lower rDNA methylation. Also *T. cacao* (angiosperm) has the lowest 35S rDNA copy number among the species analyzed, while its rDNA is densely methylated. Thus, variation in rDNA methylation levels cannot be solely explained by in rDNA copy number. Of note, *T. cacao* exhibits the lowest (5%) known global methylation levels (Vidalis et al., 2016) suggesting that rDNA does not always follow global methylation patterns.
- Methylated fraction may derive from rDNA pseudogenes. While the rDNA pseudogenes are generally rare in most plant species due to efficient concerted evolution, they may become abundant in certain seed plant groups (Harpke and Peterson, 2006; Wang et al., 2016). So far, we have no evidence that rDNA pseudogenes constitute significant proportion of rDNA arrays in bryophytes (Liu et al., 2013; Rosato et al., 2016). Out of the species analyzed, only *M. polymorpha* had significant SNPs in its 35S coding region (Rosato et al., 2016), while their pseudogenic character remains to be confirmed.
- There might be differences in auxiliary factors needed for methylation at specific loci. For example, the RNA-dependent RNA polymerase 2 involved in *de novo* methylation of *Arabidopsis* 35S rDNA through the Pol IV siRNA pathway (Preuss et al., 2008) seems to be missing in *P. patens* (A. Kovarik unpublished observation). Furthermore, compartmentalization of silencing complexes in cell nucleus also needs to be considered (Parihar et al., 2019).
- Methylation of rDNA may be dependent on an unlinked locus that seems to control expression of 35S rRNA genes in *Arabidopsis* (Chandrasekhara et al., 2016). Perhaps, a putative *in trans* controlling locus may be absent or weak in bryophytes. In this context, the chromosome epigenetic landscape seems to differ between *Arabidopsis* and *P. patens* (Lang et al., 2018).

Although differences between seed plants and bryophytes in rDNA methylation are noteworthy, it should be noted that the contrasting values found within bryophytes deserve further research. The four species for which we have available data strongly differ in life strategies, drought tolerance, and ploidy level. Thus, *P. patens* is a highly polyploid (n = 27) (though functionally haploid) (Engel, 1968) with a very ephemeral life cycle (a few weeks) and contrast with the putative haploids *P. formosum* (n = 7) and likely *D. scoparium* (n = 14), both long-lived and drought-tolerant species. Finally, *M. polymorpha* is putative haploid (n = 9), long lived, but does not show desiccation tolerance. It has been reported in mosses that drought tolerance is related to physiological aspects (Bewley, 1979) involving the conservation and reformation of polyribosomes during total desiccation (Bewley, 1972), which are active (Bewley, 1973b), allowing a fast recovery of the protein synthesis activity (Bewley, 1973a). This ecophysiological diversity of the previously discussed species does not show a clear related pattern with the patterns of rDNA methylation. However, the low number of sampled species is insufficient to clearly support or reject any hypothesis based on life cycle features.

### High Level of rDNA Chromatin Condensation Contrasts With Relatively Low Levels of H3K9m2 Heterochromatic Mark in *P. formosum* Recombinant DNA

Several cytogenetic studies indicate that moss rDNA is not exceptional with respect to its heterochromatic properties (Sone et al., 1999; Rosato et al., 2016; Orzechowska et al., 2018). Also, in two moss species (*D. scoparium* and *P. formosum*), we investigated here rDNA that exhibits high level of chromatin condensation in interphase confirming these observations. In angiosperms, the rDNA (both 35S and 5S) chromatin is dominated by heterochromatic histone H3K9m2 marks (Lawrence et al., 2004; Simon et al., 2018) believed to drive the condensation and genetic inactivity of rDNA loci in cooperation with DNA methylation. It was striking to observe that bryophyte rDNA (both 5S and 35S) shows high levels of chromatin condensation despite near absence or low level cytosine methylation and relatively low content of heterochromatic H3K9m2 marks. We cannot exclude the possibility that relatively low H3K9m2 levels are sufficient to drive formation of rDNA heterochromatin in *P. formosum*. If so, the situation in bryophytes may be reminiscent to *Drosophila* that contain very little global DNA methylation, and rDNA heterochromatin is mainly formed by histone modification marks (Peng and Karpen, 2007). However, it is also possible that the rDNA chromatin condensation in bryophytes relies on other epigenetic mechanisms, such as noncoding RNAs. In this context, transcripts derived from IGS subrepeats were identified in *P. patens* (Kirov et al., 2018). In any case, it is likely that silencing and accurate nuclear position of rDNA may function without significant cytosine methylation of units in some bryophyte species at least.

### Potential Significance of Distinct Epigenetic Features of Bryophyte rDNA Chromatin

The question arises as to the significance of distinct rDNA features in bryophyte genomes. There are several not necessarily exclusive possibilities. First, extensively hypomethylated rDNA in bryophyte genomes may be important for recombination processes that maintain high homogeneity of units. There is evidence that cytosine methylation inhibits genetic recombination in *Arabidopsis* euchromatic loci (Melamed-Bessudo and Levy, 2012). Second, the rDNA methylation deficiency that we observe here may be advantageous by preventing methylation-induced mutations in bryophytes that spend most of their life in haploid stage and in which DNA repair by homologous recombination is infrequent. These mutations severely affected rDNA homogeneity in some plant species (Buckler et al., 1997; Wang et al., 2016). Finally, demethylated or undermethylated units may be related to plant physiology potentially easing the rapid onset of biosynthesis after the inactivity during periods of dryness and freezing.

## Data Availability

The datasets generated for this study can be found in the GenBank Sequence Read Archives (project “The chromosome structure of early diverging plants,” ID: PRJNA546415).

## Author Contributions

Conceived and designed the study: AKo, RM, JR; Performed the experiments: AKr, AKo, RM, JL. Analyzed the data: RM, AKo, EM, JL; Wrote the manuscript: AKo, JR.

## Funding

The work was supported by the Czech Science Foundation (grants 19-03442S, 17-11642S, and GJ19-20530Y).

## Conflict of Interest Statement

The authors declare that the research was conducted in the absence of any commercial or financial relationships that could be construed as a potential conflict of interest.
