# Supplementary material for: Unique Epigenetic Features of Ribosomal RNA Genes (rDNA) in Early Diverging Plants (Bryophytes)
Source: Front Plant Sci. 2019 Sep 5;10:1066. doi: 10.3389/fpls.2019.01066 (PMC6739443; doi:10.3389/fpls.2019.01066)
Supplement: Supplementary file 1 [file Table_1.docx]

Table S1. Geographical location of collected plant specimens

|  | Species | Order, Family | Origin |
| --- | --- | --- | --- |
| Bryophyta | *Bryum capillare* Hedw. | Bryales, Bryaceae | Spain, Cuenca, Valdemeca, Arroyo Chico, 26-8-2012, J.A. Rosselló |
|  | *Calliergonella cuspidata* (Hedw.) Loeske | Hypnales, Hypnaceae | Spain, Cuenca, Valtablado, 25-8-2012, J.A. Rosselló |
|  | *Dicranum scoparium* Hedw. | Dicranales, Dicranaceae | 1. Czech Republic, Brno, Palackeho vrch, WGS-84: 49.2255433N, 16.5686750E, 11-11-2017, A. Krumpolcová 2. Czech Republic, Zlaté hory, WGS-84: 50.222874N, 17.367070E, E. Mikulášková |
|  | *Encalypta streptocarpa* Hedw. | Funariales, Encalyptaceae | Spain, Cuenca, Valtablado, 25-8-2012, J.A. Rosselló |
|  | *Fissidens dubius* P. Beauv. | Dicranales, Fissidentaceae | Spain, Cuenca, Valdemeca, Arroyo Chico, 26-8-2012, J.A. Rosselló |
|  | *Funaria hygrometrica* Hedw. | Funariales, Funariaceae | Czech Republic, Zlaté hory, WGS-84: 50.2178842N, 17.3635086E, E. Mikulášková |
|  | *Hypnum cupressiforme* Hedw. | Hypnales, Hypnaceae | Slovenia: Bohinj lake (Triglav National Park), 23-8-2012, J. Miravet |
|  | *Physcomitrella patens* (Hedw)*.* Bruch & Schimp | Funariales, Funariaceae | Laboratory strain ‘Gransden 2004’ |
|  | *Plagiomnium undulatum* (Hedw.) T.J. Kop. | Bryales, Miniaceae | Spain, Cuenca, Valdemeca, Arroyo Chico, 26-8-2012, J.A. Rosselló |
|  | *Pleuzorium schreberii* Hedw. | Hypnales, Hylocomiaceae | Czech Republic, Brno, Palackeho vrch (WGS-84: 49.2255433N, 16.5686750E), 20-11-2017 |
|  | *Polytrichum formosum* Hedw. | Polytrichales, Polytrichaceae | Czech Republic, Brno, Palackeho vrch (WGS-84: 49.2255433N, 16.5686750E), 20-11-2017 A. Krumpolcová |
|  | *Schistidium apocarpum* (Hedw.) Bruch & Schimp. | Grimmiales, Grimmiaceae | Spain, Cuenca, Valdemeca, Arroyo Chico, 26-8-2012, J.A. Rosselló |
|  | *Sphagnum denticulatum* Brid. | Sphagnales, Sphagnaceae | Spain, Cuenca, Valdemeca, 20-5-2012, J.A. Rosselló |
|  | *Syntrichia ruralis* (Hedw.) F. Weber & D. Mohr var. *ruralis* | Pottiales, Pottiaceae | Spain, Cuenca, Valdemeca, Arroyo Chico, 26-8-2012, J.A. Rosselló |
|  | *Thamnobryum alopecurum* (Hedw.) Gangulee | Hypnales, Neckeraceae | Slovenia: Bohinj lake (Triglav National Park), 23-8-2012, J. Miravet |
| Marchantiophyta | *Bazzania trilobata* (L.) Gray | Jungermanniales, Lepidoziaceae | Czech Republic, Brno, Palackeho vrch (WGS-84: 49.2255433N, 16.5686750E), 20-11-2017, A. Krumpolcová |
|  | *Conocephalum conicum* (L.) Underw. | Marchantiales, Conocephalaceae | Spain, Castelló, Eslida, Font Matilde, 27-8-2012, J. Miravet |
|  | *Marchantia polymorpha* L. subsp*. polymorpha* | Marchantiales, Marchantiaceae | 1. Spain, León, Campa de Mortera, 2-9-2012, F. del Egido 2. Czech Republic, Zlaté hory, WGS-84: 50.2512744N, 17.3663411E, E. Mikulášková |
|  | *Porella platyphylla* (L.) Pfeiff. | Porellales, Porellaceae | Spain, Lleida, Pico Orri, 25-7-2012, J.A. Rosselló |
| Angiosperms | *Tragopon mirus* L. | [Asterales](https://en.wikipedia.org/wiki/Asterales), Asteraceae | Czech Republic, Brno, Institute of Biophysics’s house garden, lineage 2602, orginally from the USA, Palouse.17-06-2018, R. Matyášek |
|  | *Iris versicolor* | [Asparagales](https://en.wikipedia.org/wiki/Asparagales), [Iridaceae](https://en.wikipedia.org/wiki/Iridaceae) | Czech Republic, Brno, collected in a house garden, 02-08-2010, R. Matyášek |
|  | *Nicotiana tabacum* | Solanales, Solanaceae, | Czech Republic, Brno, var. SR-1, Institute of Biophysics, grown in the greenhouse, 21-06-2015, A. Kovarik |
| Gymnosperms | *Ginkgo biloba* | Ginkgoales*,* *Ginkgoacea* | Czech Republic, Brno, collected in a city park Luzanky. WGS-84: 49.2057253N, 16.6070075E 02-07-2015, Z. Trojanek |
