# Supplementary material for: Unique Epigenetic Features of Ribosomal RNA Genes (rDNA) in Early Diverging Plants (Bryophytes)
Source: Front Plant Sci. 2019 Sep 5;10:1066. doi: 10.3389/fpls.2019.01066 (PMC6739443; doi:10.3389/fpls.2019.01066)
Supplement: Supplementary file 2 [file Table_2.docx]

Table S2. Basic statistics of high-throughput sequencing carried out in this study

| **species** | **1C [Mb]^1^** | **DNA** | **Reads^2^** | **Coverage^3^** | **Q20[%]** | **GC[%]** |
| --- | --- | --- | --- | --- | --- | --- |
| ***P. formosum*** | 523 | native | 100,169,766 | 28.7 | 94.6 | 48.5 |
|  | 523 | bisulfite | 100,968,538 | 29.0 | 96.1 | 26.8 |
| ***D. scoparium*** | 711 | native | 17,428,486 | 3.7 | 94.2 | 43.1 |
|  | 711 | bisulfite | 14,144,810 | 3.0 | 97.9 | 30.0 |

^1^ Genome size. Data from the Plant DNA C-values Database (Bennet and Leitch, 2012)

^2^ Number of 150 bp-long Illumina reeds

^3^ Calculated from as follows: Number of Reads * Read length (bp) */Genome size (Mb) *10^6^.
