# Supplementary material for: Unique Epigenetic Features of Ribosomal RNA Genes (rDNA) in Early Diverging Plants (Bryophytes)
Source: Front Plant Sci. 2019 Sep 5;10:1066. doi: 10.3389/fpls.2019.01066 (PMC6739443; doi:10.3389/fpls.2019.01066)
Supplement: Supplementary file 3 [file Table_3.docx]

Table S3. List of species, read archives and number of bisulfite reads used in methylation analyses

| Species | SRA  identification | reads  total 18S rDNA 5S rDNA | | | 35S rDNA |
| --- | --- | --- | --- | --- | --- |
| *Arabidopsis thaliana* | SRR1984607 | 17,014,092 | 101,526 | 167,594 | n.d. |
| *Brassica napus* | SRR7763971 | 19,934,519 | 129,378 | 7,724 | n.d. |
| *Dicranum scoparium* | SRR8707091^1^ | 14,144,810 | 40,300 | 1,271 | 154,401 |
| *Marchantia polymorpha* | SRR2070688 | 27,593,360 | 191,083 | 7,431 | 551,977 |
| *Oryza sativa* | SRR1026165 | 93,046,051 | 155,130 | 126,069 | n.d. |
| *Physcomitrella patens* | SRR1824569 | 58,770,374 | 363,468 | 17,083 | 1,070,662 |
| *Polytrichum formosum* | SRR8707304^1^ | 50,484,269 | 293,994 | 3,085 | 787,802 |
| *Solanum lycopersicum* | SRR346428 | 14,061,618 | 239,917 | 7,357 | 754,277 |
| *Theobroma cacao* | SRR3286313 | 13,077,678 | 13,788 | 1,045 | 33,550 |

^1^ Sequencing data are from this work
