# Supplementary material for: Unique Epigenetic Features of Ribosomal RNA Genes (rDNA) in Early Diverging Plants (Bryophytes)
Source: Front Plant Sci. 2019 Sep 5;10:1066. doi: 10.3389/fpls.2019.01066 (PMC6739443; doi:10.3389/fpls.2019.01066)
Supplement: Supplementary file 4 [file Table_4.docx]

Table S4. The positions and size of rDNA subregions in assembled rDNA units

| **species** | **region** | **Start** | **End** | **Length** |
| --- | --- | --- | --- | --- |
| ***P. formosum*** | **ETS(partial** | 1 | 900 | 900 |
|  | **18S** | 901 | 2714 | 1814 |
|  | **ITS1** | 2715 | 3244 | 530 |
|  | **5.8 S** | 3245 | 3401 | 157 |
|  | **ITS2** | 3402 | 3691 | 290 |
|  | **26S** | 3692 | 6878 | 3187 |
|  | **IGS1** | 6879 | 7354 | 476 |
|  | **5S** | 7355 | 7474 | 120 |
|  | **IGS2(partial)** | 7475 | 9044 | 1570 |
|  | **Whole contig** | 1 | 9044 | 9044 |
| ***D. scoparium*** | **ETS(partial** | 1 | 222 | 222 |
|  | **18S** | 223 | 2047 | 1825 |
|  | **ITS1** | 1826 | 2311 | 264 |
|  | **5.8 S** | 2312 | 2468 | 157 |
|  | **ITS2** | 2469 | 2802 | 334 |
|  | **26S** | 2803 | 6194 | 3392 |
|  | **IGS1** | 6195 | 6572 | 378 |
|  | **5S** | 6573 | 6692 | 120 |
|  | **IGS2(partial)** | 6693 | 7003 | 311 |
|  | **Whole contig** | 1 | 7003 | 7003 |
