# Supplementary material for: Unique Epigenetic Features of Ribosomal RNA Genes (rDNA) in Early Diverging Plants (Bryophytes)
Source: Front Plant Sci. 2019 Sep 5;10:1066. doi: 10.3389/fpls.2019.01066 (PMC6739443; doi:10.3389/fpls.2019.01066)
Supplement: Supplementary file 5 [file Table_5.docx]

Table S5. rDNA copy number calculated from high-throughput data

| **Species** | **1C^1^**  **[Mb]** | **Total reads** | **Mapped reads**  **18S^5^  5S^6^** | | **GP[%]^2^**  **18S^5^ 5S^6^** | | **GS[kb]^3^**  **18S^5^ 5S^6^** | | **CN^4^/1C**  **18S^5^ 5S^6^** | |
| --- | --- | --- | --- | --- | --- | --- | --- | --- | --- | --- |
| ***P. formosum*** | 523 | 50,084,883 | 95,070 | 6,358 | 0.190 | 0.013 | 993 | 66 | 552 | 553 |
| ***D. scoparium*** | 711 | 13,941,031 | 39,177 | 2,772 | 0.281 | 0.020 | 1,998 | 141 | 1,110 | 1,178 |
| ***P. patens*** | 518 | 30,224,053 | 86,403 | 8,702 | 0.286 | 0.029 | 1,481 | 149 | 823 | 1,243 |
| ***M. polymorpha*** | 287 | 141,348,695 | 2,313,304 | 104,173 | 1.637 | 0.074 | 4,697 | 212 | 2,609 | 1,763 |
| ***A. thaliana*** | 135 | 27,163,511 | 126,840 | 62,916 | 0.467 | 0.232 | 630 | 313 | 350 | 2,606 |
| ***B. napus*** | 1,182 | 24,352,996 | 90,859 | 11,245 | 0.373 | 0.046 | 4,410 | 546 | 2,450 | 4,548 |
| ***S. lycopersicum*** | 1,002 | 13,765,308 | 74,927 | 5,148 | 0.544 | 0.037 | 5,454 | 375 | 3,030 | 3,123 |
| ***O. sativa*** | 489 | 6,459,525 | 15,648 | 1,543 | 0.242 | 0.024 | 1,185 | 117 | 658 | 973 |
| ***T. cacao*** | 416 | 11,930,374 | 15,300 | 1,178 | 0.128 | 0.010 | 533 | 41 | 296 | 342 |

^1^ Data from the Plant DNA C-values Database (Bennet and Leitch, 2012)

^2^ GP - genome proportion (Material and Methods)

^3^ GS - genome space (Material and Methods)

^4^ copy number

^5^ 1.8 kb 18S rDNA region

^6^ 0.12 kb 5S rDNA region
