# Supplementary material for: Unique Epigenetic Features of Ribosomal RNA Genes (rDNA) in Early Diverging Plants (Bryophytes)
Source: Front Plant Sci. 2019 Sep 5;10:1066. doi: 10.3389/fpls.2019.01066 (PMC6739443; doi:10.3389/fpls.2019.01066)
Supplement: Supplementary file 6 [file Table_6.docx]

Table S6. CHIP results calculated from three independent experiments

|  |  |  |  |  |  |  |  |  |  |  |  |  |  |  |  |
| --- | --- | --- | --- | --- | --- | --- | --- | --- | --- | --- | --- | --- | --- | --- | --- |
| BR^1^ | TR^2^ | ct_(H3)_ |  | ct |  |  |  | d(ct) = ct-ct_(H3)_ | | |  | relative abundance = 2^-d(ct)^ | | | |
|  |  | H3 |  | H3K9me2 | | H3K4me3 | | H3K9me2 | | H3K4me3 | | H3K9me2 | | H3K4me3 | |
|  |  | PF^3^ | TM^4^ | PF | TM | PF | TM | PF | TM | PF | TM | PF | TM | PF | TM |
| A | 1 | 20.6 | 18.7 | 20.8 | 14.1 | 23.1 | 20.7 | 1.3 | -2.3 | 2.5 | 3.5 | 0.4 | 4.9 | 0.2 | 0.1 |
|  | 2 | 21.4 | 17.2 | 21.9 | 14.9 | 23.1 | 20.2 | -1.8 | -5.5 | 0.2 | -1.2 | 3.5^(^ | 45.3 | 0.9 | 2.3 |
|  | 3 | 22.6 | 19.6 | 21.7 | 14.1 | 22.8 | 18.4 |  |  |  |  |  |  |  |  |
|  | aver.-A | 21.5 | 18.5 | 21.5 | 14.4 | 23.0 | 19.8 | 0.0 | -4.1 | 1.5 | 1.3 | 1.0 | 17.1 | 0.4 | 0.4 |
|  | SD-A | 0.7 | 0.9 | 0.4 | 0.4 | 0.1 | 0.9 | 0.8 | 1.0 | 0.7 | 1.3 |  |  |  |  |
| B | 1 | 27.5 | nd | 28.0 | 20.8 | 28.9 | 24.7 | 1.2 | -2.8 | 3.7 | 0.3 | 0.4 | 7.0 | 0.1 | 0.8 |
|  | 2 | 27.4 | 25.3 | 28.3 | 20.8 | 30.8 | 23.9 | -0.2 | -4.5 | 1.3 | -1.4 | 1.1 | 22.6 | 0.4 | 2.6 |
|  | 3 | 27.1 | 24.4 | 27.3 | 21.6 | 28.8 | 24.3 |  |  |  |  |  |  |  |  |
|  | aver.-B | 27.3 | 24.9 | 27.9 | 21.1 | 29.5 | 24.3 | 0.6 | -3.8 | 2.2 | -0.6 | 0.7 | 13.9 | 0.2 | 1.5 |
|  | SD-B | 0.2 | 0.5 | 0.4 | 0.4 | 0.9 | 0.3 | 0.4 | 0.6 | 0.9 | 0.6 |  |  |  |  |
| C | 1 | nd | nd | 21.5 | 24.8 | nd | 32.7 | -1.8 | -3.7 | -0.9 | 4.2 | 3.5 | 13.0 | 1.9 | 0.1 |
|  | 2 | 23.3 | 29.7 | 20.8 | nd | 20.7 | 30.3 | -3.1 | -4.9 | -3.2 | 0.6 | 8.6 | 29.9 | 9.2 | 0.7 |
|  | 3 | 23.9 | 28.5 | 20.9 | nd | 22.4 | 31.3 |  |  |  |  |  |  |  |  |
|  | aver.-C | 23.6 | 29.1 | 21.1 | 24.8 | 21.6 | 31.4 | -2.5 | -4.3 | -2.0 | 2.3 | 5.7 | 19.7 | 4.0 | 0.2 |
|  | SD-C | 0.3 | 0.6 | 0.3 | 0.0 | 0.9 | 0.8 | 0.4 | 0.6 | 0.9 | 1.0 |  |  |  |  |
| aver.^5^ |  |  |  |  |  |  |  |  |  |  |  | 2.5 | 16.9 | 1.5 | 0.7 |
| SD |  |  |  |  |  |  |  |  |  |  |  | 2.2 | 2.0 | 1.6 | 0.5 |

^1^ BR- biological replicates were represented by three different nuclei isolates (A, B and C)

^2^ Parallel samples analysed by quantitative PCR

^3^ *Polytrichum formosum*

^4^ *Tragopogon mirus*

^5^ Vales used to construct a graph in Figure 5

Yellow shading - average values, green shading – maximal values, blue shading – minimal values.
