# Supplementary material for: Unique Epigenetic Features of Ribosomal RNA Genes (rDNA) in Early Diverging Plants (Bryophytes)
Source: Front Plant Sci. 2019 Sep 5;10:1066. doi: 10.3389/fpls.2019.01066 (PMC6739443; doi:10.3389/fpls.2019.01066)
Supplement: Supplementary file 7 [file DataSheet_1.pdf]

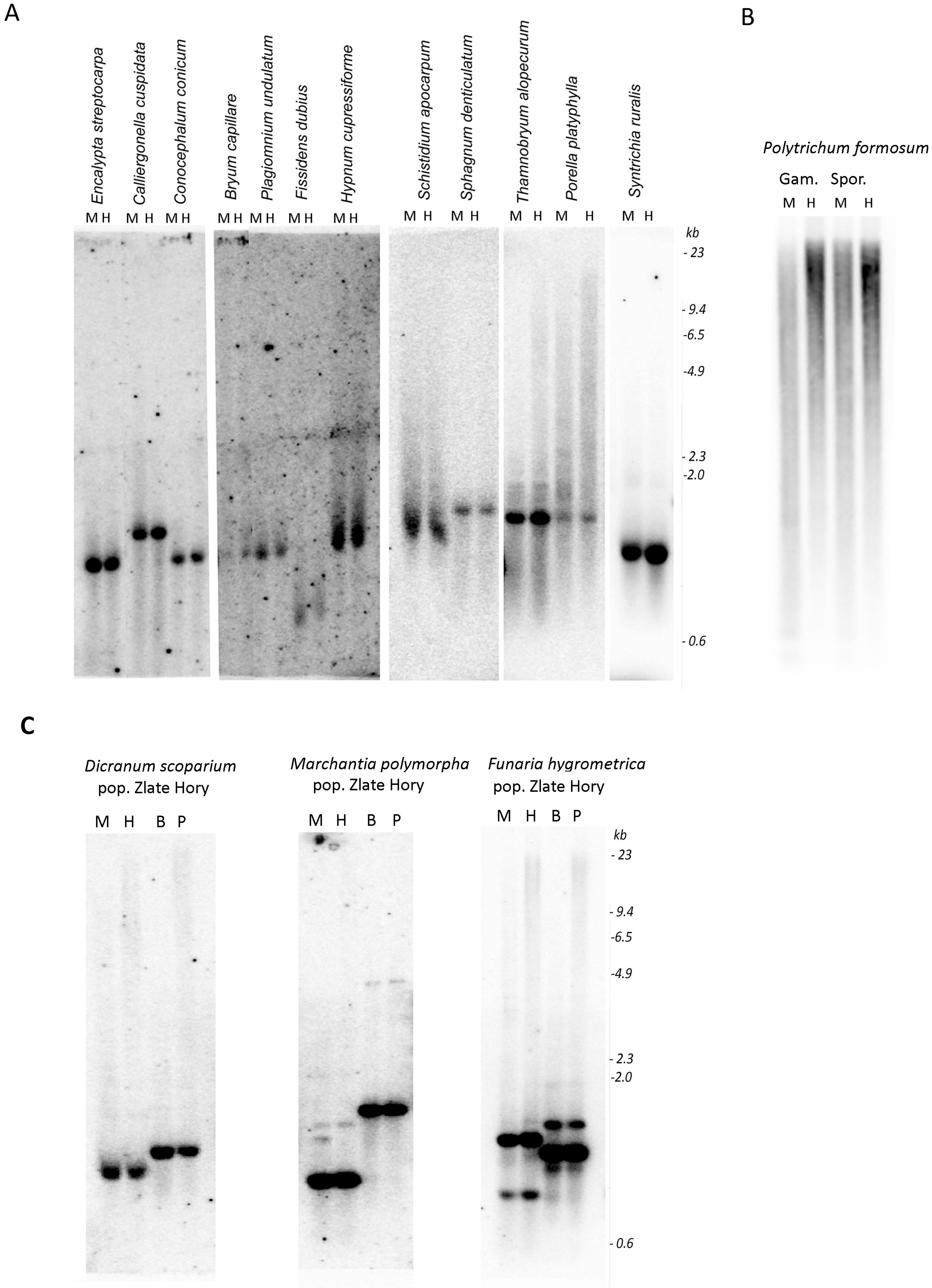

Figure S1 Southern blot hybridization of the 26S rDNA (A-C), PforCL1 (B) probes to the *Msp*I (M),*Hpa*II (H), *Bst*NI (B) and *Psp*GI (P) - digested genomic DNAs. In (C) analysis of three species from Zlate Hory populations (Czech Republic) is shown. Gam. - gametophyte, Spor. - sporophyte.
