# Supplementary material for: Unique Epigenetic Features of Ribosomal RNA Genes (rDNA) in Early Diverging Plants (Bryophytes)
Source: Front Plant Sci. 2019 Sep 5;10:1066. doi: 10.3389/fpls.2019.01066 (PMC6739443; doi:10.3389/fpls.2019.01066)
Supplement: Supplementary file 8 [file DataSheet_2.pdf]

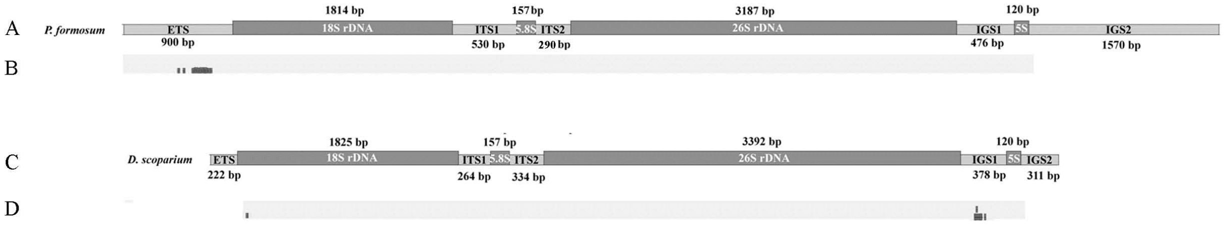

Figure S2 Diagrams of assembled *P. formosum* (A) and *D. scoparium* (C) rDNA units. Positions of polymorphic sites are shown in (B, D). There were 40 and 19 single nucleotide variants (SNV) in the rDNA units in *P. formosum* and *D. scoparium*, respectively.
