# Supplementary material for: Unique Epigenetic Features of Ribosomal RNA Genes (rDNA) in Early Diverging Plants (Bryophytes)
Source: Front Plant Sci. 2019 Sep 5;10:1066. doi: 10.3389/fpls.2019.01066 (PMC6739443; doi:10.3389/fpls.2019.01066)
Supplement: Supplementary file 9 [file DataSheet_3.pdf]

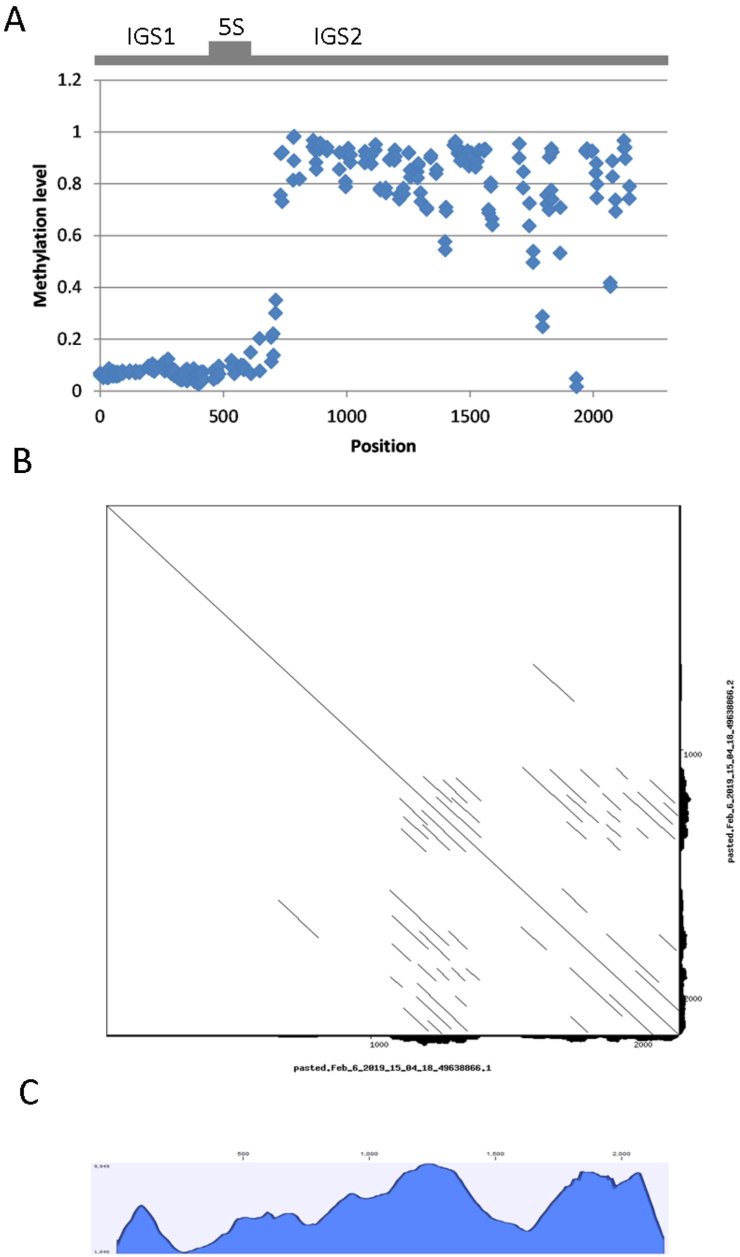

Figure S3. An illustration of potential problems associated with mapping of bisulfite reads to repetitive subregions of IGS. (A) Methylation plot versus IGS1-5S-IGS2 subregions of *the P. formosum* rDNA unit. (B) Dot plot self to self comparison of the IGS1-5S-IGS2 sequence. Note, highly repetitive region immediately downstream of the 5S rRNA gene. (C) Read coverage graph along the IGS1-5S-IGS2 subregion.
