# Supplementary material for: Unique Epigenetic Features of Ribosomal RNA Genes (rDNA) in Early Diverging Plants (Bryophytes)
Source: Front Plant Sci. 2019 Sep 5;10:1066. doi: 10.3389/fpls.2019.01066 (PMC6739443; doi:10.3389/fpls.2019.01066)
Supplement: Supplementary file 10 [file DataSheet_4.pdf]

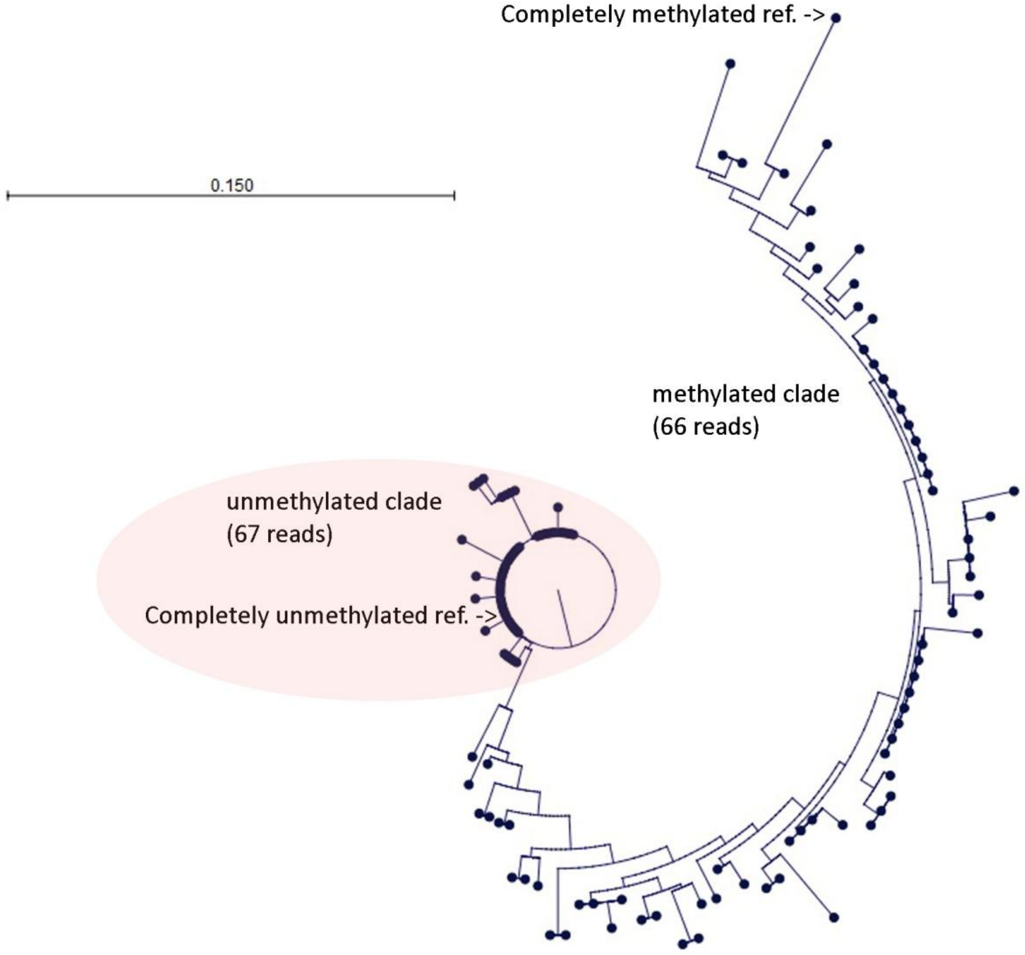

Figure S4 Phylogenetic tree constructed from 135 bisulfite reads derived from the *D. scoparium* 18S rDNA subregion located between positions 773 and 891 of the 35S-5S rDNA contig (Supplementary Table S4). The 119 bp-long sequences were aligned with the completely methylated (native molecule) and non-methylated (in which all Cs were converted into Ts) reference sequences. Note, a good separation of clades containing fully methylated and non-methylated sequences.
