# Supplementary material for: Unique Epigenetic Features of Ribosomal RNA Genes (rDNA) in Early Diverging Plants (Bryophytes)
Source: Front Plant Sci. 2019 Sep 5;10:1066. doi: 10.3389/fpls.2019.01066 (PMC6739443; doi:10.3389/fpls.2019.01066)
Supplement: Supplementary file 11 [file DataSheet_5.pdf]

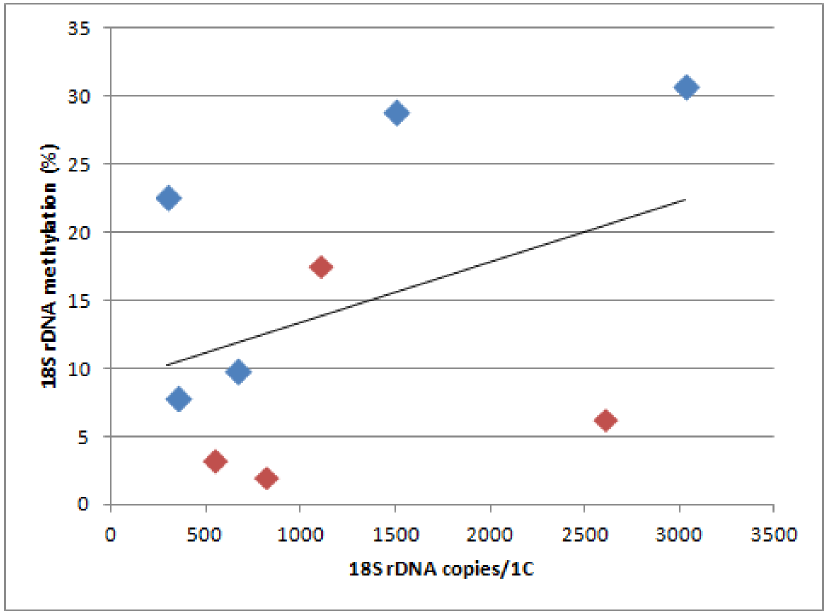

Slope	Intercep	R^2	Mean(X)	SS(x)	S(x*y)	SS(y)	F	DFn	DFd	P
1.637501	-125.795	**0.804164**	1214.27	7827055	12816812	26098590	28.74419	1	7	**0.001051**

Figure S5 A relationship between 18S rDNA copy number and its methylation levels. For the species analysed and the datasets, see Fig. 2 and Supplementary Table S5. Blue diamonds - angiosperms; red diamonds- bryophytes.
